# Trends in genitourinary cancer mortality in the United States: analysis of the CDC-WONDER database 1999–2020

**DOI:** 10.3389/fpubh.2024.1354663

**Published:** 2024-06-17

**Authors:** Yahia Ghazwani, Mohammad Alghafees, Mahammed Khan Suheb, Areez Shafqat, Belal Nedal Sabbah, Tarek Ziad Arabi, Adhil Razak, Ahmad Nedal Sabbah, Marwan Alaswad, Wael AlKattan, Abderrahman Ouban, Saleha Abdul Rab, Kenan Abdulhamid Shawwaf, Mohammad AlKhamees, Ahmed Alasker, Abdullah Al-Khayal, Bader Alsaikhan, Abdulmalik Addar, Lama Aldosari, Abdullah A. Al Qurashi, Ziyad Musalli

**Affiliations:** ^1^College of Medicine at King Saud bin Abdulaziz University for Health Sciences (KSAU-HS), Ministry of the National Guard Health Affairs, Riyadh, Saudi Arabia; ^2^Division of Urology, King Abdulaziz Medical City, Ministry of the National Guard Health Affairs, Riyadh, Saudi Arabia; ^3^King Abdullah International Medical Research Centre, Ministry of the National Guard Health Affairs, Riyadh, Saudi Arabia; ^4^St. Luke’s Aurora Hospital, Milwaukee, WI, United States; ^5^College of Medicine, Alfaisal University, Riyadh, Saudi Arabia; ^6^Department of Cardiothoracic Surgery, Mayo Clinic, Phoenix, AZ, United States; ^7^Department of Urology, McGill University, Montreal, QC, Canada; ^8^Department of Urology, King Fahad University Hospital, Khobar, Saudi Arabia; ^9^College of Medicine, King Saud bin Abdulaziz University for Health Sciences at the National Guards, Jeddah, Saudi Arabia

**Keywords:** prostate cancer, bladder cancer, kidney cancer, testicular cancer, United States, mortality, gender disparities, racial disparities

## Abstract

**Introduction:**

Sociodemographic disparities in genitourinary cancer-related mortality have been insufficiently studied, particularly across multiple cancer types. This study aimed to investigate gender, racial, and geographic disparities in mortality rates for the most common genitourinary cancers in the United States.

**Methods:**

Mortality data for prostate, bladder, kidney, and testicular cancers were obtained from the Centers for Disease Control and Prevention (CDC) WONDER database between 1999 and 2020. Age-adjusted mortality rates (AAMRs) were analyzed by year, gender, race, urban–rural status, and geographic region using a significance level of *p* < 0.05.

**Results:**

Overall, AAMRs for prostate, bladder, and kidney cancer declined significantly, while testicular cancer-related mortality remained stable. Bladder and kidney cancer AAMRs were 3–4 times higher in males than females. Prostate cancer mortality was highest in black individuals/African Americans and began increasing after 2015. Bladder cancer mortality decreased significantly in White individuals, Black individuals, African Americans, and Asians/Pacific Islanders but remained stable in American Indian/Alaska Natives. Kidney cancer-related mortality was highest in White individuals but declined significantly in other races. Testicular cancer mortality increased significantly in White individuals but remained stable in Black individuals and African Americans. Genitourinary cancer mortality decreased in metropolitan areas but either increased (bladder and testicular cancer) or remained stable (kidney cancer) in non-metropolitan areas. Prostate and kidney cancer mortality was highest in the Midwest, bladder cancer in the South, and testicular cancer in the West.

**Discussion:**

Significant sociodemographic disparities exist in the mortality trends of genitourinary cancers in the United States. These findings highlight the need for targeted interventions and further research to address these disparities and improve outcomes for all populations affected by genitourinary cancers.

## Introduction

Cancer stands as one of the top three leading causes of death in the United States (US), second only to heart disease (1). As the US population ages, the burden of cancer on public health and, by extension, the economy will only increase. Among the different cancer types, genitourinary cancers have shown a steady increase in incidence, particularly in developed countries like the US, imposing a substantial burden in terms of morbidity, mortality, and health expenditures (2). Despite this prevalence and variability across factors like age, gender, race, ethnicity, and geography, there remains a poor understanding of the different outcomes in diverse patient groups. Previous studies have focused on individual cancer types such as prostate, bladder, and kidney cancer (3–5).

Recent advancements in surveillance methods and access to extensive datasets have allowed researchers to explore the temporal patterns of these cancers. For instance, a recent study utilized the Surveillance, Epidemiology, and End Results (SEER) to evaluate cross-sectional mortality rates, country-level geographic patterns, and short- and long-term temporal trends in the incidence and mortality of the four genitourinary cancers (prostate, bladder, kidney, and testes) (6).

Our study utilizes the Center for Disease Control and Prevention (CDC) WONDER database to investigate differences in the age-adjusted mortality rates (AAMRs) for these four genitourinary cancers based on sex, age, race, state, urban/rural residence, and region in the US from 1999 to 2020. By shedding light on these trends, we hope to empower decision-makers with targeted actions to reduce cancer-related fatalities. Furthermore, our work seeks to advocate for developing and sharing large-scale global databases to foster further research on genitourinary tumors.

## Methods

### Study setting and population

This study analyzed mortality data from the Centers for Disease Control and Prevention (CDC) WONDER database between 1999 and 2020 for prostate, kidney, bladder, and testicular cancer (7). Cases were identified and collected using the 10th revision of the International Statistical Classification of Diseases (ICD-10) codes. Death certificates with prostate, kidney, bladder, and testicular cancer listed as the main cause of death were examined. Sensitivity analysis was conducted using specific ICD codes for underlying causes of death. Age groups were categorized as 15–24, 25–34, 35–44, 45–54, 55–64, 65–74, 75–84, and 85 years and above. The authors deemed this project to be exempt from institutional review board approval as deidentified and publicly available data were used. All methods employed in our study were conducted strictly per the Strengthening the Reporting of Observational Studies in Epidemiology (STROBE) guidelines (8). All data collection, analysis, and interpretation were performed under our institution’s established ethical standards and protocols and in compliance with international research standards.

### Data abstraction

Data on the size of the population, year of death, location of death, demographic characteristics, National Center for Health Statistics urban–rural classification, and geographic region of the patient’s residence were collected. Demographic parameters included gender, age group, and race category as defined by the US Census Bureau (Black/African American, White, Asian and Pacific Islander, and American Indian and Alaska Native) (9).

### Statistical analysis

Trends in prostate, kidney, bladder, and testicular cancer crude and age-adjusted mortality rates (AAMRs) from 1999 to 2020 were stratified by year, gender, race, urban–rural status, and geographic region reported per 10,000 population with 95% CIs. Crude mortality rates were calculated by dividing the number of prostate, kidney, bladder, and testicular cancer cases by the US population for the corresponding year. AAMRs were determined by standardizing prostate, kidney, bladder, and testicular cancer deaths to the US population in the year 2000. The Joinpoint Regression Program evaluated yearly trends in prostate, kidney, bladder, and testicular cancer mortalities by calculating annual percent changes (APCs) with 95% CIs in AAMRs. This method identifies significant changes in AAMR over time and considers increasing or decreasing trends significant if the slope describing the change in mortality differs significantly from zero (*p* < 0.05).

## Results

### Prostate cancer

**Figure 1:**
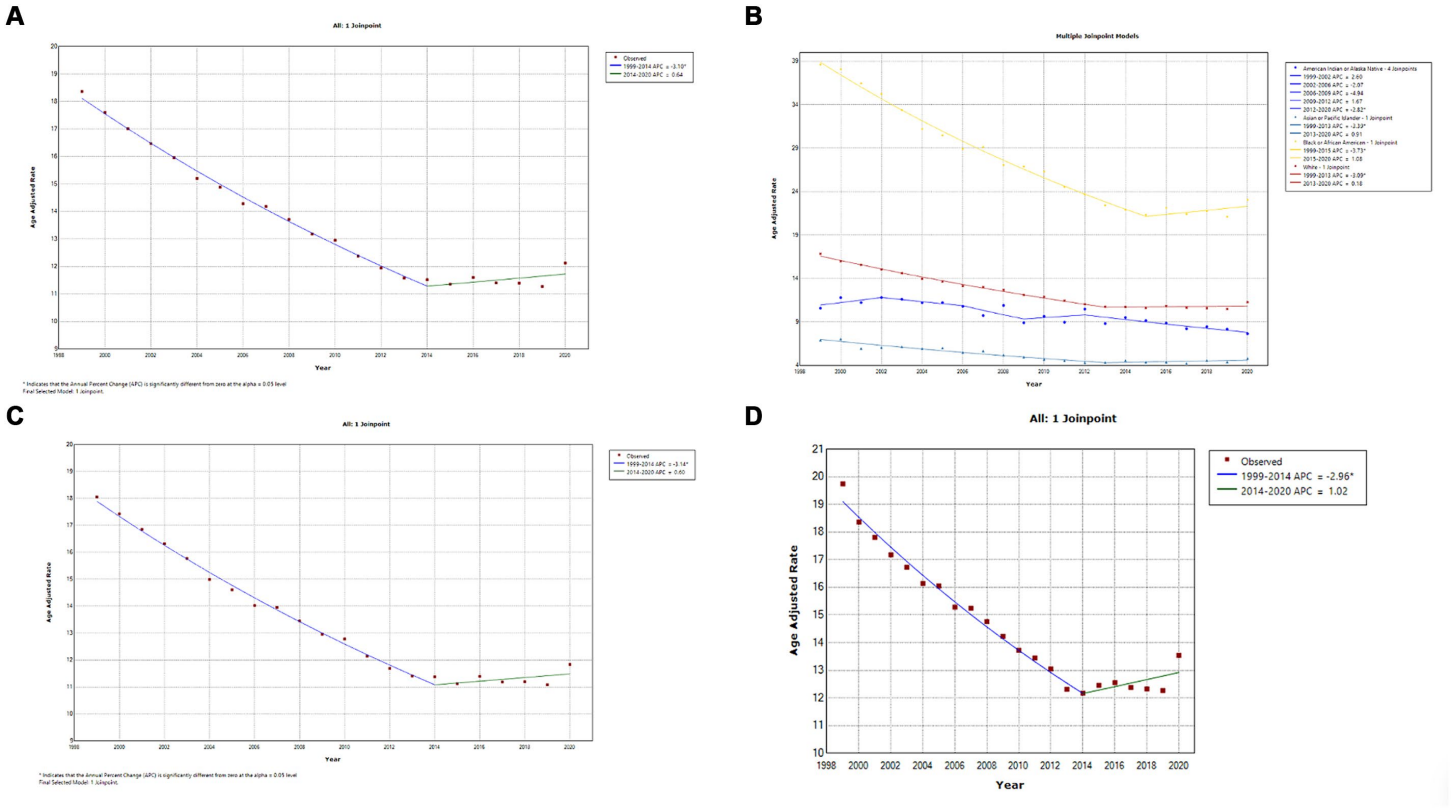
**(A)** Overall temporal mortality trend for prostate cancer showing a significant decline between 1999 and 2020. **(B)** Racial differences in prostate cancer mortality in the United States. **(C)** Temporal trends of prostate cancer mortality in metropolitan areas. **(D)** Temporal trends of prostate cancer mortality in non-metropolitan areas.

**Figure 2:**
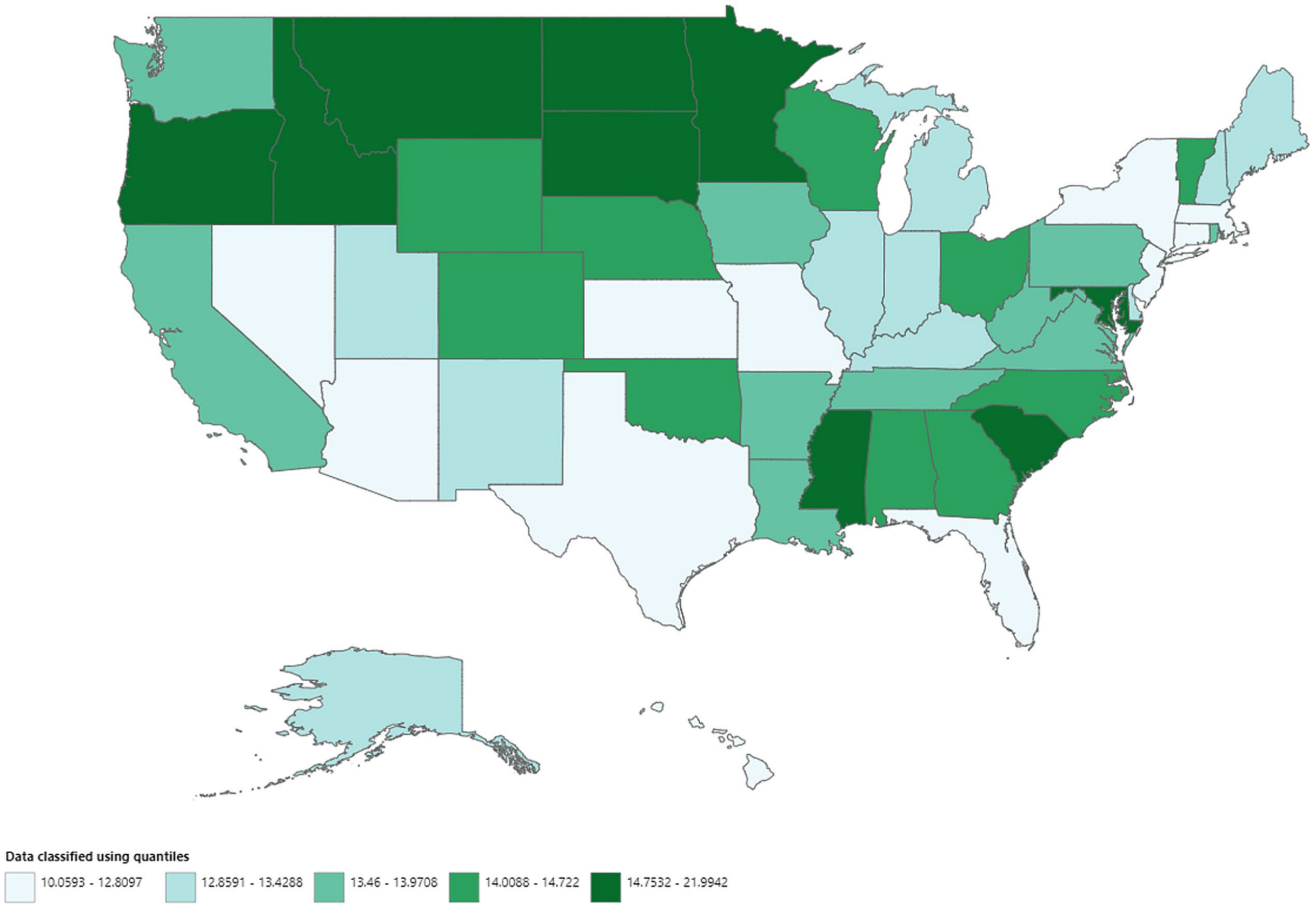
Geographic disparities in prostate cancer mortality.

A total of 606,059 deaths from prostate cancer were recorded in adults (aged 25–85 years) between 1999 and 2020. Overall, the AAMR for prostate cancer-related decreased significantly from 18.4 (95% CI, 18.1–18.5) in 1999 to 11.6 (95% CI, 11.4–11.7) in 2014, an APC of −3.1 (95% CI, −3.3 to −2.9; *p* < 0.001). Overall, AAMR for prostate cancer stabilized from 2014 to 2020 (APC, 0.6; 95% CI, −0.1 to 1.4; *p* = 0.096; Figure 1A; Supplementary Table 1A). Blacks/African Americans had the highest AAMR throughout the study period, followed by Whites, American Indian/Alaska Natives, and Asian/Pacific Islanders in descending order (Figure 1B; Supplementary Table 1B). The AAMR declined most significantly in Blacks/African Americans from 1999 to 2015 (APC, −3.7; 95% CI, −4.0 to −3.5; *p* < 0.001) but has stabilized since 2015 (APC, 1.1; 95% CI, −0.4 to 2.6; *p* = 0.142). Whites and Asians/Pacific Islanders followed a similar trend, displaying significant declines in AAMR from 1999 to 2013 that have since stabilized. The AAMR of prostate cancer in American Indian/Alaska Natives showed a variable trend from 1999 to 2012 but, unlike the other races, declined significantly from 2012 to 2020 (APC, −2.8; 95% CI, −4.1 to −1.5; *p* = 0.001) (Figure 1B; Supplementary Table 1B). The AAMR of prostate cancer declined significantly in both metropolitan and non-metropolitan areas from 1999 to 2014 but is now increasing (Figures 1C,D, respectively; Supplementary Tables 1D,E, respectively). Geographically, the highest AAMRs were observed in the Midwest (AAMR, 13.7; 95% CI, 13.7–13.8), followed by the West (13.6; 95% CI, 13.5–13.6), South (13.5; 95% CI, 13.4–13.5), and Northeast (12.9; 95% CI, 12.8–12.9) in descending order (Figure 2; Supplementary Figure 1). The District of Columbia and Mississippi had the highest prostate cancer mortality rates, whereas the lowest rates were seen in Hawaii, Florida, Arizona, and Massachusetts (Supplementary Figure 2).

### Bladder cancer

**Figure 3:**
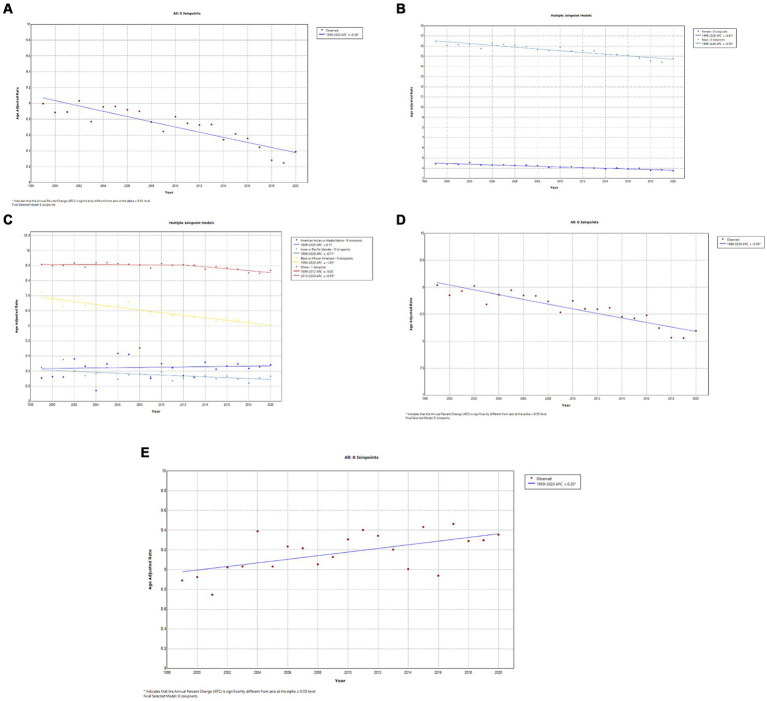
**(A)** Overall temporal mortality trend for bladder cancer in the United States between 1999 and 2020. **(B)** Gender disparities in bladder cancer mortality. **(C)** Racial disparities in bladder cancer mortality. **(D)** Bladder cancer mortality in metropolitan areas. **(E)** Bladder cancer mortality in non-metropolitan areas.

**Figure 4:**
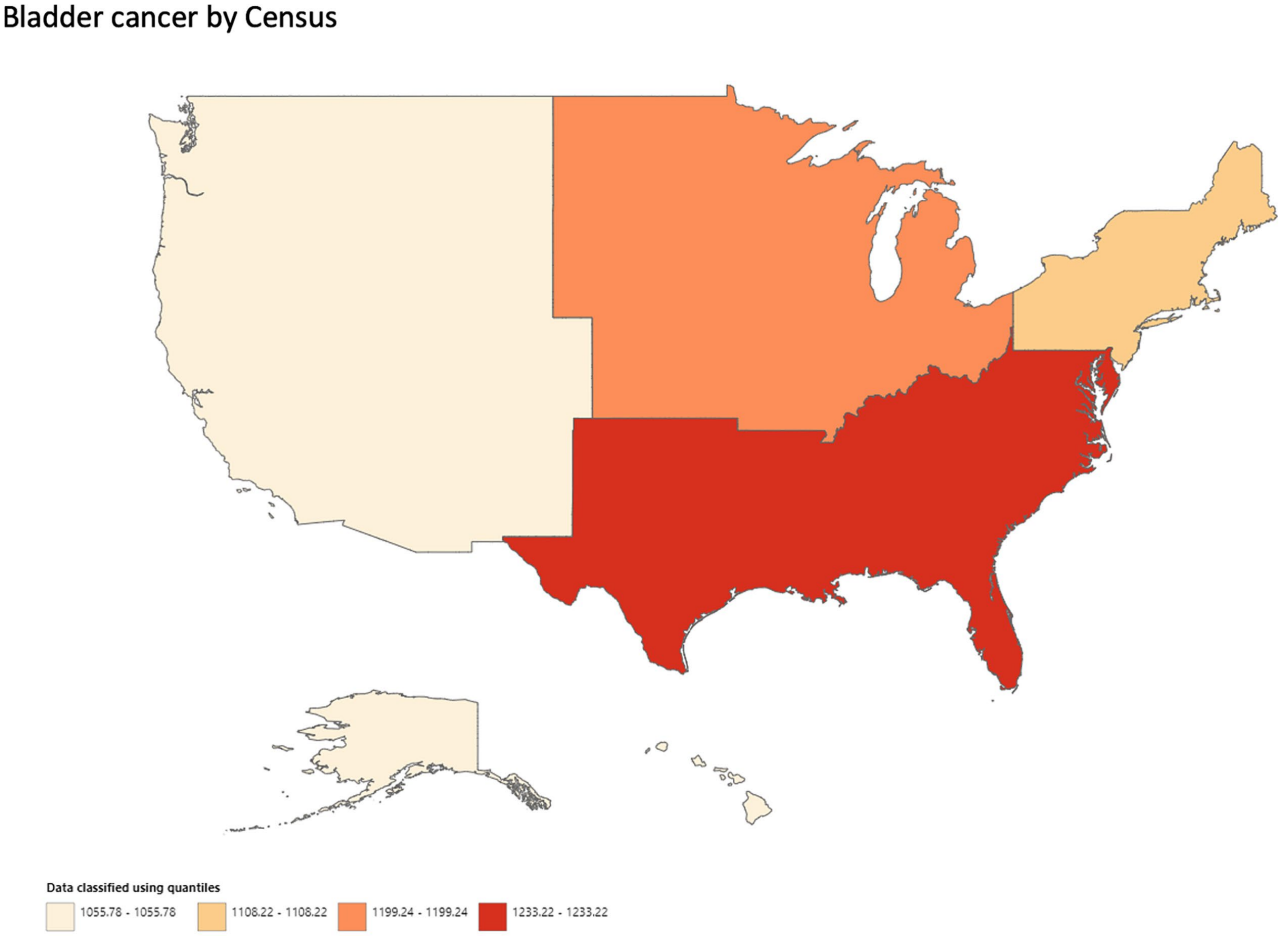
Geographic disparities in bladder cancer mortality.

A total of 413,696 deaths were reported from bladder cancer in adults (25–85 years) between 1999 and 2020. Overall, bladder cancer AAMR declined steadily and significantly from 1999 to 2020 (APC, −0.4; 95% CI, −0.5 to −0.3; *p* < 0.001) (Figure 3A; Supplementary Table 2A). Men had approximately a 4-fold higher AAMR as compared to women throughout the study period, but AAMRs of bladder cancer declined significantly in both men (APC, −0.6; 95% CI, −0.6 to −0.5; *p* < 0.001) and women (APC, −0.8; 95% CI, −0.9 to −0.7; *p* < 0.001) 1999 and 2020. (Figure 3B; Supplementary Table 2B). Whites had the highest AAMR, followed by Blacks/African Americans, American Indian/Alaska Natives, and Asian/Pacific Islanders in descending order (Figure 3C; Supplementary Figure 3C). Temporally, AAMR plateaued in White individuals between 1999 and 2012 (APC, 0.0; 95% CI, −0.2 to 0.1) but then declined significantly from 2012 to 2020 (APC, −0.6; 95% CI, −0.9 to −0.2; *p* < 0.002). In contrast, Black/African Americans (APC, −1.0; 95% CI, −1.2 to −0.9; *p* < 0.001) and Asian/Pacific Islanders (APC, −0.7; 95% CI, −1.1 to −0.3) showed a steady decline in AAMR from 1999 to 2020. AAMRs in American Indians/Alaska Natives remained stable, with no change in the mortality rate (Figure 3C) (Supplementary Table 3C). The AAMR in metropolitan areas declined significantly from 1999 to 2020 (APC, −0.5; 95% CI, −0.6 to −0.4; *p* < 0.001), while mortality increased in non-metropolitan areas (APC, 0.2; 95% CI, 0.1–0.3; *p* = 0.003) (Figures 3D,E; Supplementary Tables 2D,E). Geographically, the highest AAMR was observed in the South, followed by the Midwest, Northeast, and West (Figure 4; Supplementary Figure 3). The states with the highest bladder cancer-related mortality rates were Maine, Vermont, and Rhode Island, with the lowest rates in Hawaii and Utah (Supplementary Figure 4).

### Kidney cancer

**Figure 5:**
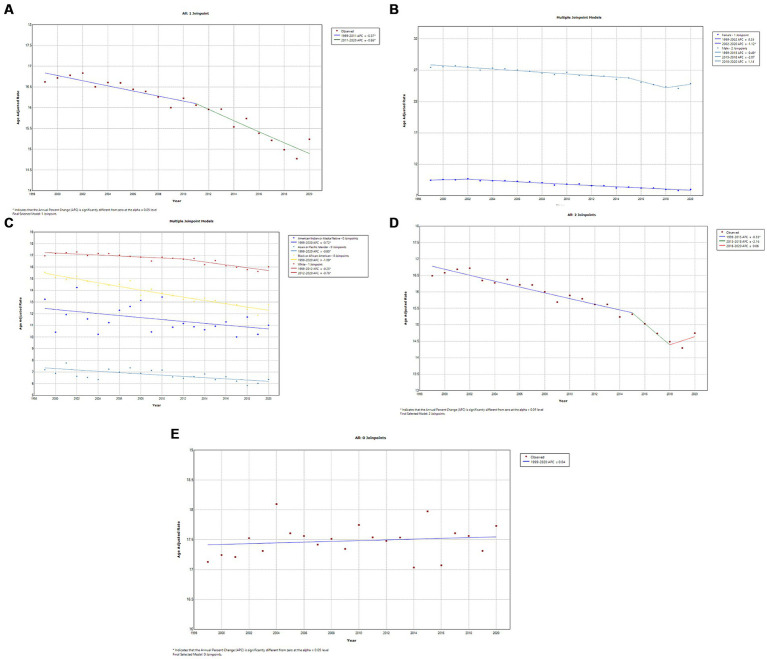
**(A)** Overall temporal mortality trend for kidney cancer in the United States between 1999 and 2020. **(B)** Gender disparities in kidney cancer mortality. **(C)** Racial disparities in kidney cancer mortality. **(D)** Kidney cancer mortality in metropolitan areas. **(E)** Kidney cancer mortality in non-metropolitan areas.

**Figure 6:**
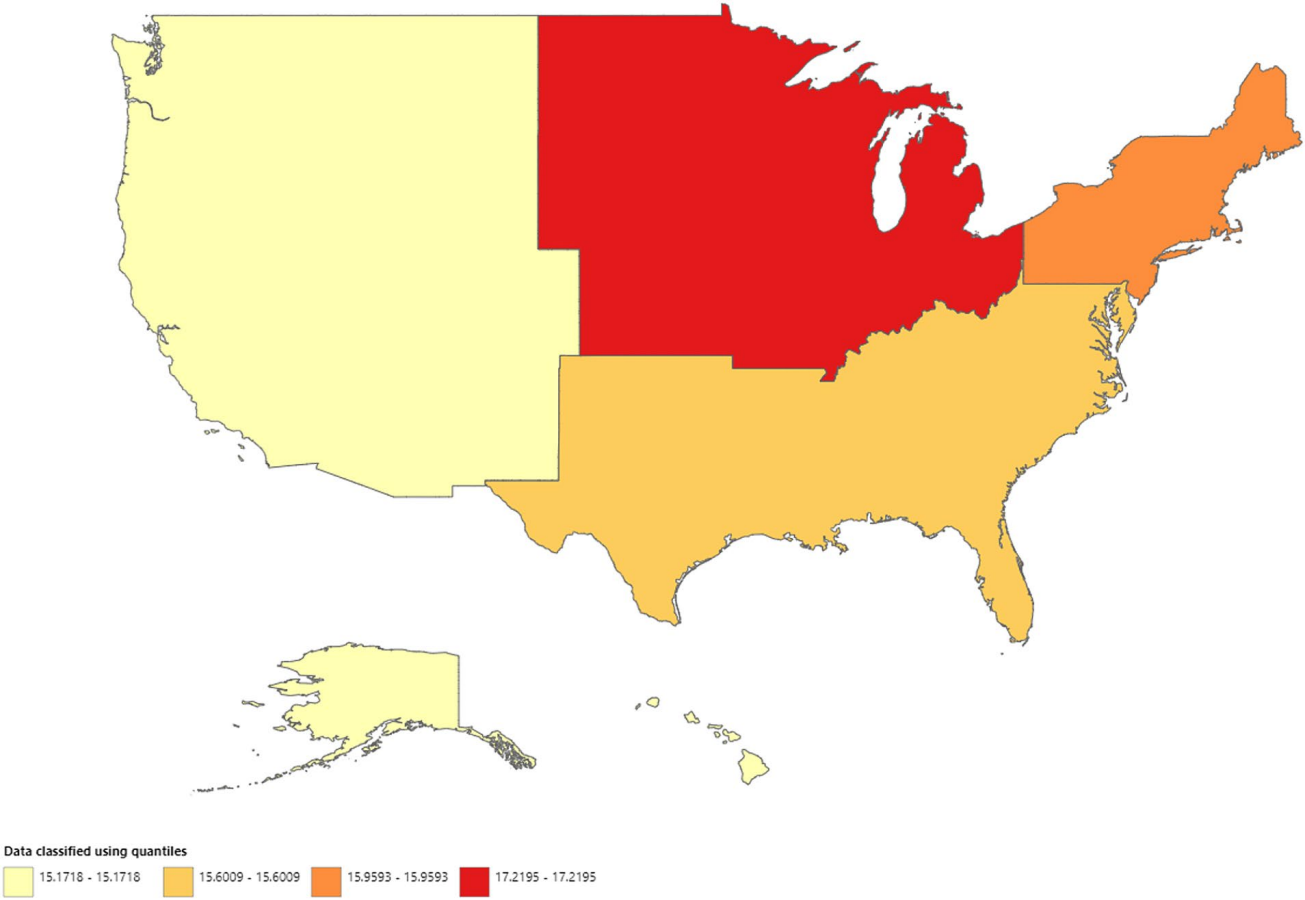
Geographic disparities in kidney cancer mortality.

A total of 282,096 deaths occurred from kidney cancer in adults (25–84 years) between 1999 and 2020. Temporally, AAMR declined slowly from 1999 to 2011 (APC, −0.4; 95% CI, −0.6 to −0.2; *p* = 0.001) and then steeply from 2011 to 2020 (APC, −0.6; 95% CI, −1.1 to −0.6; *p* < 0.001) (Figure 5A; Supplementary Table 3A). Kidney cancer-related AAMR was approximately 4-fold higher in men compared to women and displayed gender-specific temporal trends (Figure 5B; Supplementary Table 3B). Mortality rates in men declined steadily from 1999 to 2015 (APC, −0.5; 95% CI, −0.6 to −0.4; *p* < 0.001) and statistically insignificant changes until 2020. In contrast, women displayed a steady decline from 2002 to 2020 (APC, −1.1; 95% CI, −1.2 to −1.0; *p* < 0.001) (Figure 5B; Supplementary Table 3B). White individuals had the highest kidney cancer-related mortality rates, which decreased slowly from 1999 to 2012 (APC, −0.3; 95% CI, −0.4 to −0.1; *p* = 0.011) and then at a greater rate from 2012 to 2020 (APC, −0.8; 95% CI, −1.1 to −0.4; *p* < 0.001) (Figure 5C; Supplementary Table 3C). In contrast, Blacks/African Americans (APC, −1.1; 95% CI, −1.2 to −0.9; *p* < 0.001) and American Indians/Alaska Natives (APC, −0.7; 95% CI, −1.3 to −0.1; *p* = 0.023) exhibited steeper declines from 1999 to 2020. Asians/Pacific Islanders had the lowest overall AAMR and declined steadily (APC, −0.8; 95% CI, −1.2 to −0.4; *p* < 0.001) (Figure 5C; Supplementary Table 3C). Kidney cancer-related mortality declined steadily in metropolitan areas until 2018, whereas it remained relatively stable in non-metropolitan regions (Figures 5D,E, respectively; Supplementary Tables 3D,E, respectively). Geographically, the highest AAMR was observed in the Midwest, followed by the South, West, and Northeast (Figure 6; Supplementary Figure 5). The mortality rate by state was highest in Maine, Vermont, and West Virginia, whereas Hawaii and Utah boasted the lowest mortality rates (Supplementary Figure 6).

### Testicular cancer

**Figure 7:**
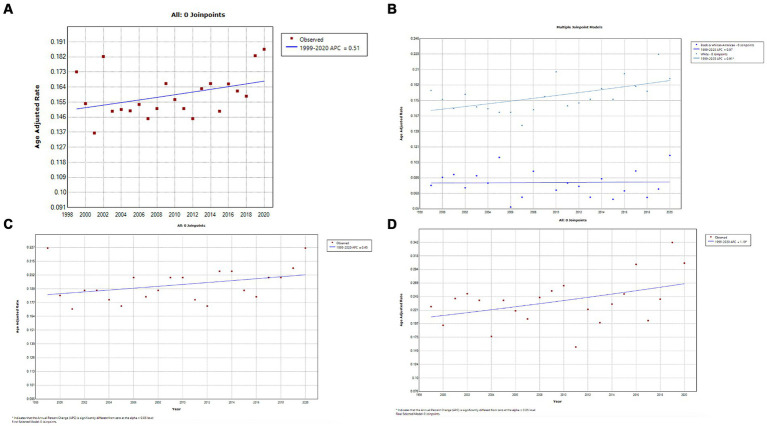
**(A)** Overall temporal mortality trend for testicular cancer in the United States between 1999 and 2020. **(B)** Racial disparities in testicular cancer mortality. **(C)** Testicular cancer mortality in metropolitan areas. **(D)** Testicular cancer mortality in non-metropolitan areas.

**Figure 8:**
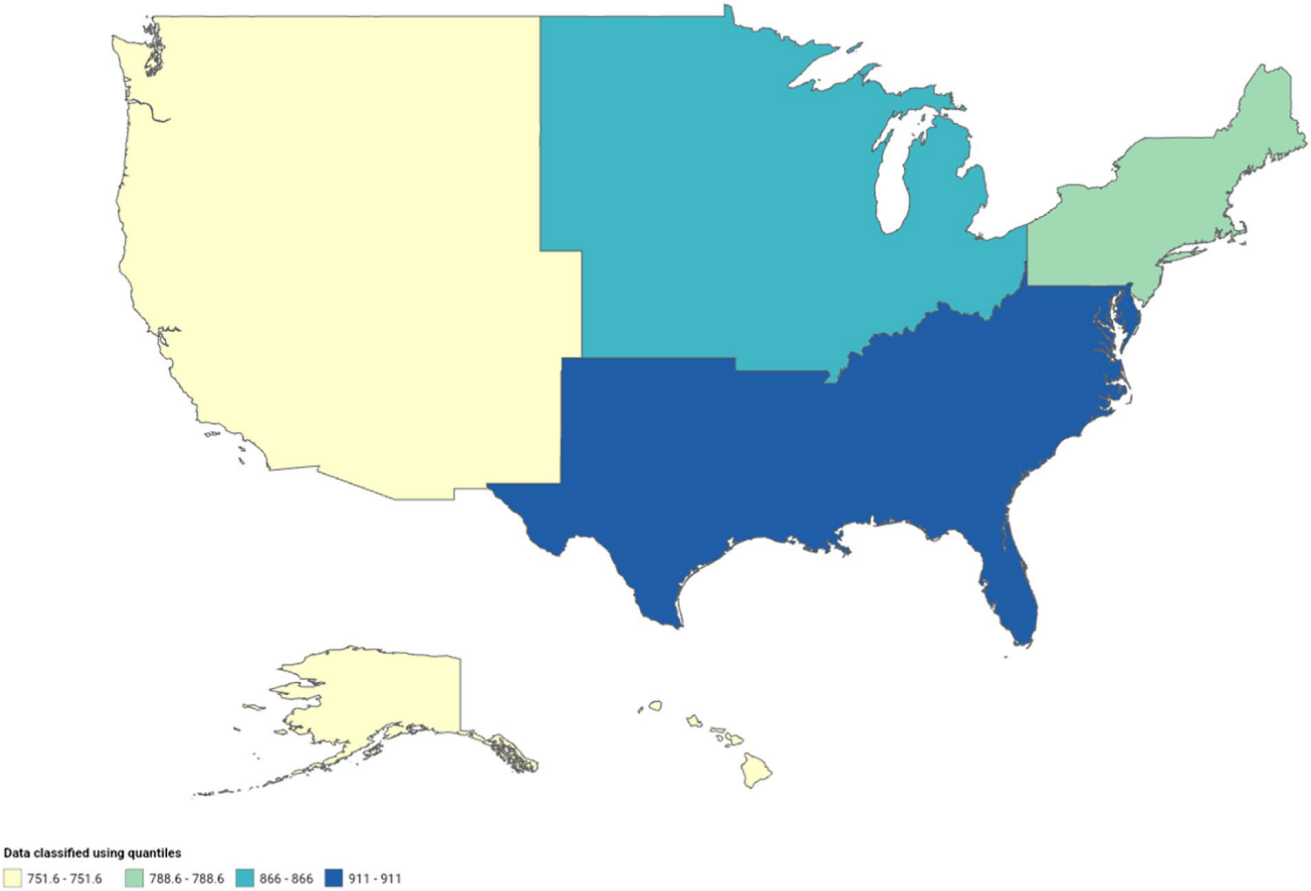
Geographic disparities in testicular cancer mortality.

A total of 11,024 deaths were recorded from testicular cancer in adults (25–84 years) between 1999 and 2020. Temporally, AAMRs for testicular remained stable from 1999 to 2020 (APC, 0.5; 95% CI, 0.0–1.1; *p* = 0.069) (Figure 7A; Supplementary Table 4A). AAMRs for American Indians/Alaskan Natives and Asian/Pacific Islanders were suppressed due to small data values; the CDC-WONDER database suppresses information when counts fall below a “cut-off” value. For the remaining races, AAMRs for Whites showed a steady and statistically significant increase between 1999 and 2020 (APC, 0.9; 95% CI, 0.3–1.5; *p* = 0.005), whereas the AAMR for Blacks/African Americans remained stable (APC, 0.1; 95% CI, −1.3 to 1.5; *p* = 0.917) (Figure 7B; Supplementary Table 4B). Testicular cancer-related AAMR significantly increased in non-metropolitan areas, whereas changes in metropolitan areas were not statistically significant (Figures 7C,D, respectively; Supplementary Tables 4C,D, respectively). Geographically, the highest AAMR was observed in the West, Midwest, Northeast, and South in descending order (Figure 8; Supplementary Figure 7). Oklahoma stood out has having the highest testicular cancer mortality rate, followed by Arizona and California, whereas Virginia and Delaware had the lowest mortality rates (Supplementary Figure 8).

## Discussion

Leveraging the nationwide CDC WONDER database, we observed temporal variations in genitourinary cancer mortality based on gender, race, and geography in the US from 1999 to 2020. The observed decline in the AAMRs of prostate, bladder, and kidney cancer may be attributable to the increased uptake of screening methods, such as serum prostate-specific antigen (PSA) levels and diagnostic imaging, and advances in the treatment of genitourinary cancers that have prolonged overall survival. Testicular cancer was an important exception, demonstrating a stable overall AAMR during the study period and a statistically significant increase in Whites. Nonetheless, relative to all genitourinary cancers, testicular cancer maintained the lowest AAMR and mortality burden, a testament to its favorable treatment response that leads to long-term survival in most patients (10).

The factors underpinning the disproportionate representation of Blacks/African Americans in prostate cancer incidence and mortality are still being studied. Individuals of African descent are more likely to harbor genetic variants associated with a higher risk of prostate cancer development (11). Studies have also indicated a potential racial bias in the US that puts Black men at higher risks of receiving inadequate treatment and having lower survival rates compared to White patients with prostate cancer (12–14).

The introduction of prostate cancer screening by PSA played a significant role in increasing the diagnoses of prostate cancer in its early stages during the 2000s and early 2010s, reflected in our findings as a declining mortality rate. The US Preventative Services Task Force’s (USPTF) recommendation to limit PSA-based screening in the early 2010s coincided with an increase in the incidence of advanced-stage prostate cancer (3, 15). Accordingly, we observed a rise or stabilization in overall prostate cancer AAMR after 2014, most notable among Black, White, and Asian/Pacific Islanders; American Indians/Alaskan Natives continued their steady decline. Even after the revised 2018 USPTF recommendation suggesting that high-risk individuals—namely Blacks/African Americans and those with a family history of prostate cancer—discuss the benefits and drawbacks of PSA-based screening to allow for shared decision-making, we did not observe a decline in prostate cancer AAMR in the Black/African American population.

Bladder cancer demonstrated a significantly higher AAMR in men compared to women, consistent with recent US data (2, 6). While smoking is a well-known risk factor for bladder cancer and is more prevalent among males, the higher incidence and mortality of bladder cancer in men compared to women in the US cannot be solely attributed to this factor (16, 17). Sex hormone effects on the bladder, sex-specific responses to carcinogens, and differential exposure of the urothelium to carcinogens have been suggested as additional factors behind this gender disparity (16). Regarding race, white individuals demonstrated a higher AAMR for bladder cancer as compared with other races, consistent with the higher incidence of bladder cancer in Whites (6). The reasons behind this are also poorly understood and cannot be fully explained by lifestyle differences like smoking, as it is more prevalent among Blacks/African Americans (18). However, a subset analysis assessing bladder cancer mortality rates between races stratified by gender demonstrated comparable mortality rates for bladder cancer between black and white women in the US despite black women having a 36% lower incidence of bladder cancer (19). To explain this, Black patients with muscle-invasive bladder cancer are less likely to receive optimal treatment and being black is also an independent risk factor for increased 90-day mortality for bladder cancer (19). These findings indicate a persisting racial bias in bladder cancer treatment and survival that must be addressed.

Kidney cancer is twice as common in men than in women (20). This was reflected in our study as a higher AAMR in males than in females. Females also demonstrate better survival outcomes from kidney cancer, and global data has reported a decrease in kidney cancer mortality in females by 11.3% between 1990 and 2013, while a 9.9% increase was observed in males (21). Our US-based data showed that AAMR decreased in both genders, with a steeper annual decline in females and a recent statistically non-significant increase in AAMR in males between 2018 and 2020. American Indian/Alaska Natives have the highest incidence of kidney cancer, perhaps due to a higher prevalence of risk factors such as smoking and higher BMI in this group (22). However, we observed the highest AAMRs of kidney cancer in White people. A recent study utilizing the CDC WONDER database to report disparities in kidney cancer AAMR between 1999 and 2020 also demonstrated the highest AAMR in White individuals. Still, American Indians/Alaska Natives initially had the highest AAMR in 1999 (5). This contrasts our findings, where White individuals displayed the highest AAMR throughout the study period. These discrepant results may be due to the CDC WONDER database being constantly updated, including additional records or corrections not present during the prior study.

The higher cancer mortality burden and a slower decline in genitourinary cancer-related mortality in rural versus urban communities are well documented (23). Our results showing a statistically significant increase in bladder cancer AAMR and a stable kidney cancer AAMR in rural populations are concerning and have recently been reproduced (23). Reasons for poorer cancer outcomes in rural areas include unequal access to cancer screening facilities, a failure to control lifestyle risk factors such as obesity and smoking, and poorer health literacy among rural residents (24). Cancer patients from rural communities are also more prone to missing appointments, having to travel longer distances to get treatment, being diagnosed at a later stage, being treated at smaller treatment facilities and not at specialist centers, and having fewer opportunities for enrollment into clinical trials as compared to urban neighborhoods, hence resulting in poorer outcomes (25).

On geographic disparities, our findings are consistent with a recent SEER database analysis demonstrating a higher prostate cancer mortality in Midwestern and Western states despite its relatively low incidence in these regions (6). This is unlikely to be because of inequalities in access to healthcare, as the Western states have some of the highest percentages of insurance coverage. While the incidence of kidney cancer is highest in Appalachia and other Southern states, we observed its mortality is highest in the Midwest (6). Although bladder cancer incidence is highest in the Northeast, widely believed to be due to arsenic contaminating water wells in this region (26), we observed the highest mortality of bladder cancer in the South. We also identified clusters of high mortality for specific genitourinary cancers in specific states. Importantly, Mokdad et al. (27) showed that clusters of high mortality existed for kidney, bladder, prostate, and testicular cancer in counties that may be masked when only looking at overall AMMR or at the level of the state. Potential explanations for these variations in mortality can be divided into distinct aspects of cancer epidemiology: a varying prevalence of risk factors (e.g., lifestyle factors such as obesity, diabetes and smoking, and genetic risk factors that negatively impact prognosis); different levels of success of public health screening and prevention policies; varying degrees of access to cancer treatment and structural factors like the healthcare infrastructure itself and insurance coverage; and different cancer treatment strategies (6, 28). The public health response to these findings should involve improving cancer screening opportunities for malignancies where screening benefits have been shown, controlling risk factors and comorbidities associated with genitourinary cancers, and improving access to the standard-of-care cancer therapies.

An examination of different data sources enriches our understanding of cancer epidemiology. A recent study by Schafer et al. (6) examined incidence and mortality trends of genitourinary cancers in the US, spanning a period similar to the present analysis. This study utilized the SEER database, a population-based nationwide database that offers detailed information on cancer incidence and survival and includes comprehensive historical and demographic data crucial for ascertaining temporal trends in cancer incidence and mortality (29). However, the SEER database covers only approximately 34% of the US population, potentially limiting the generalizability of its findings. Another database, the National Cancer Database (NCDB), contains data from hospital registries from Commission on Cancer-accredited facilities, capturing about 70% of the country’s newly diagnosed cancer cases (30). While the NCDB contains detailed clinical information that can be used to ascertain the quality of cancer treatment and response rates, this database is limited to CoC-accredited facilities and, therefore, may not be fully representative of cancer outcomes, especially across racial and ethnic minorities and those who do not have access to these facilities.

The SEER database is often the database of choice when assessing sociodemographic disparities in cancer incidence and mortality trends. We utilized the CDC-WONDER database, which, on the one hand, is easily accessible and provides comprehensive national coverage by incorporating mortality data from all US states and territories, but on the other hand, may not capture the same level of detail regarding cancer-specific mortality and sociodemographic data as the SEER database. However, our study utilizing the CDC WONDER database is the first direct comparison of different national databases, and our findings are largely concordant with the recent findings of Schafer et al., who utilized the SEER database (6).

The present study highlights several critical areas for future research. The increase in prostate cancer AAMR after the change in PSA-based screening guidelines particularly underscores the importance of informed decision-making on PSA-based screening, particularly in Black individuals, as well as the identification of more genetic variants and lifestyle factors that enable an individualized screening strategy (31). Interpreting PSA levels considering race may also offer valuable insights (32). Our study also advocates for adopting the American Urological Association’s PSA screening guidelines, which recommends routine screening of at-risk individuals beginning at age 40–45 years, as well as offering baseline screening and frequent screening intervals in all men starting at age 45 years (33). We reproduced rural–urban disparities in genitourinary cancer mortality, which have remained unchanged over the past two decades. We observed a discrepancy in genitourinary cancer mortality in different geographic regions of the United States, which was not concordant with incidence data reported from previous studies and required further studies to identify the root causes.

Our study’s strengths include utilizing a comprehensive, nationwide database over a two-decade period, which allowed us to assess changes in mortality in light of technological and treatment advancements and changing cancer screening guidelines. However, limitations include inaccuracies in reporting data, such as misclassification of gender, race, and cause of death from death certificates, which could mislead the mortality data for American Indian/Alaska Natives and Asian/Pacific Islanders because of their relatively low representation in national databases. We did not identify gender disparities in the AAMR of genitourinary cancers within the studied racial groups, nor did we address racial disparities regarding genitourinary cancer mortality in rural/non-metropolitan areas. Thirdly, we did not compare mortality trends between Hispanic and non-Hispanic ethnicities.

For future studies, improving the representation of varying races and ethnicities in cancer databases is essential to obtain a more accurate understanding of incidence and mortality trends. For example, American Indian/Alaskan Natives and Asian/Pacific Islanders currently exhibit the lowest incident genitourinary cancer capture rates in the National Cancer Database. In contrast, capture rates are highest among Whites (34). Moreover, our study period does not cover the COVID-19 pandemic, which significantly impacted cancer care and disproportionately affected the treatment of cancer patients belonging to racial minority groups and those in rural areas, as well as increased the prevalence of obesity in US adults (35). Therefore, we recommend that future studies document mortality trends during this period.

## Conclusion

Our study provides insights into the temporal trends and disparities in the death rates from genitourinary cancers in the US. Over the past 2 decades, the significant decline in mortality rates of genitourinary cancers represents a tremendous advance. Still, we demonstrated considerable gender, racial, and geographic disparities in genitourinary cancer mortality rates that continue to exist today. Understanding the burden of risk factors contributing to genitourinary cancer incidence and mortality across diverse patient groups and geographic regions will be essential for devising effective public health policies to better these trends.

## Data availability statement

The original contributions presented in the study are included in the article/Supplementary material, further inquiries can be directed to the corresponding author.

## Ethics statement

Ethical approval was not required for the study involving humans in accordance with the local legislation and institutional requirements. Written informed consent to participate in this study was not required from the participants or the participants’ legal guardians/next of kin in accordance with the national legislation and the institutional requirements.

## Author contributions

YG: Writing – original draft, Validation, Supervision, Project administration, Methodology, Investigation, Data curation, Conceptualization. MAlg: Writing – review & editing, Writing – original draft, Supervision, Project administration, Methodology, Investigation, Data curation, Conceptualization. MS: Writing – original draft, Methodology, Investigation, Data curation, Conceptualization. ASh: Writing – review & editing, Supervision, Writing – original draft, Methodology, Investigation, Data curation, Conceptualization. BS: Validation, Project administration, Writing – review & editing, Writing – original draft, Supervision, Methodology, Investigation, Data curation, Conceptualization. TA: Writing – review & editing, Writing – original draft, Supervision, Project administration, Methodology, Investigation, Data curation, Conceptualization. AR: Writing – review & editing, Writing – original draft, Visualization, Investigation, Conceptualization. ASa: Writing – review & editing, Writing – original draft, Methodology, Investigation, Data curation, Conceptualization. MAla: Writing – original draft, Methodology, Investigation, Data curation, Conceptualization. WA: Writing – original draft, Investigation, Conceptualization. AO: Writing – original draft, Investigation, Conceptualization. SA: Writing – original draft, Investigation, Conceptualization. KS: Writing – original draft, Investigation. MAlK: Writing – original draft, Investigation. AhA: Writing – original draft, Project administration, Methodology, Investigation, Conceptualization. AbA: Writing – original draft, Supervision, Project administration, Methodology, Investigation, Conceptualization. BA: Writing – original draft, Supervision, Methodology, Investigation, Conceptualization. AAd: Writing – original draft, Methodology, Investigation, Conceptualization. LA: Writing – original draft, Investigation. AAA: Writing – original draft, Investigation, Data curation. ZM: Writing – review & editing, Writing – original draft, Supervision, Project administration, Methodology, Investigation, Data curation, Conceptualization.
