# Supplementary material for: Trends in genitourinary cancer mortality in the United States: analysis of the CDC-WONDER database 1999–2020
Source: Front Public Health. 2024 Jun 17;12:1354663. doi: 10.3389/fpubh.2024.1354663 (PMC11223728; doi:10.3389/fpubh.2024.1354663)

**Supplementary Table 1a.** Temporal mortality trend of prostate cancer in the US.


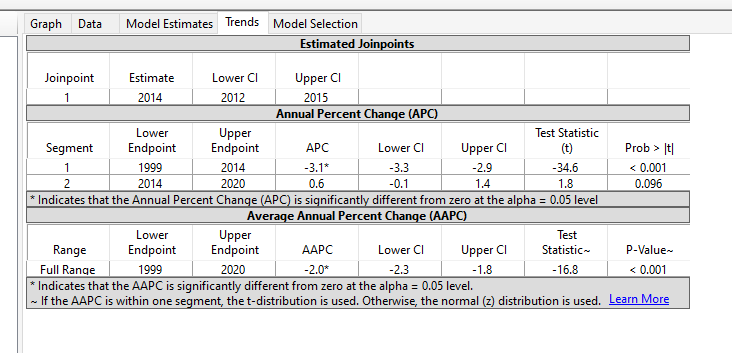


**Supplementary Table 1b.** Temporal trends of prostate cancer by race.


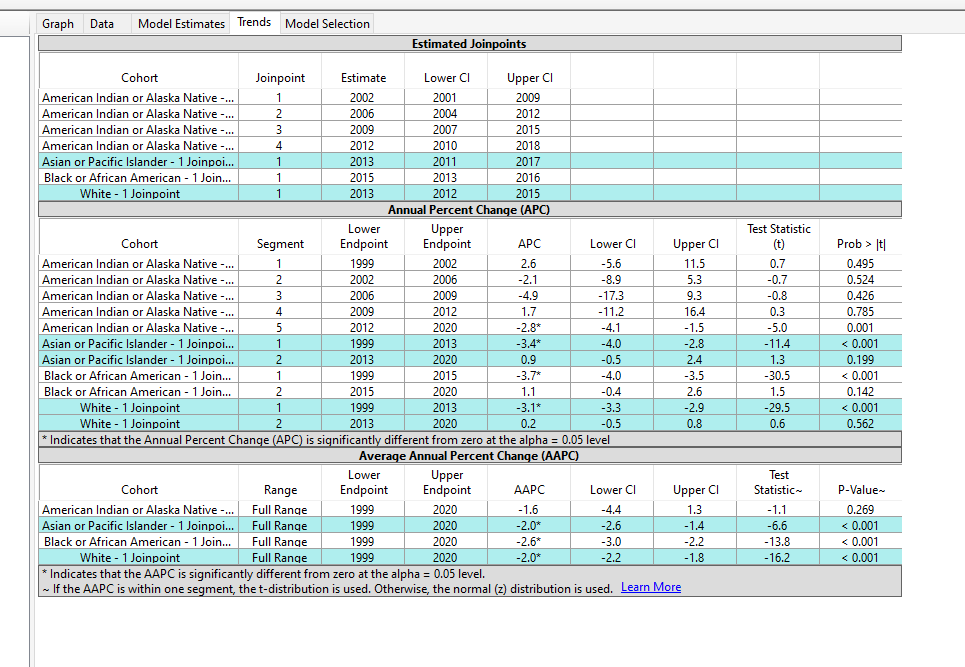


**Supplementary Table 1c.** Temporal trends of prostate cancer in metropolitan areas.


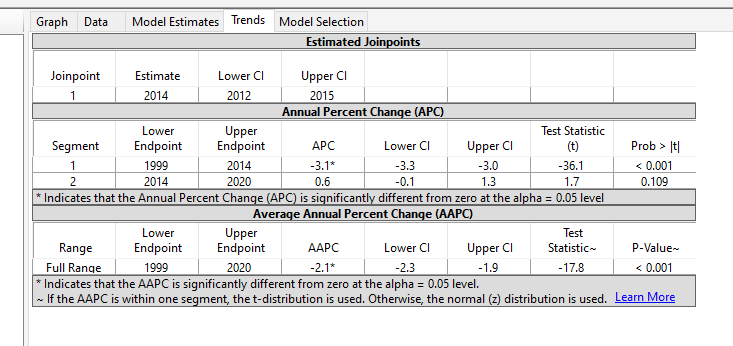


**Supplementary Table 1d.** Temporal trends of prostate cancer in non-metropolitan areas.


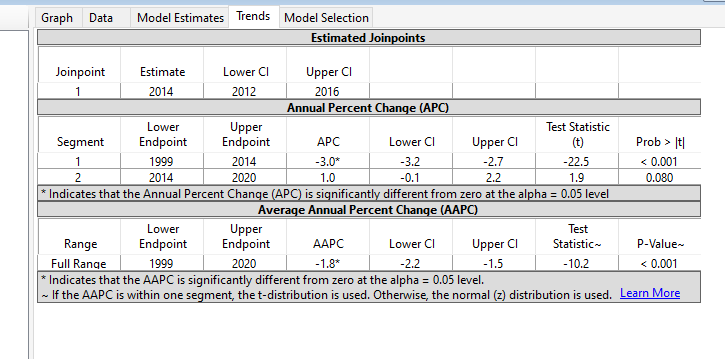


**Supplementary Table 2a.** Temporal mortality trend of bladder cancer in the US.


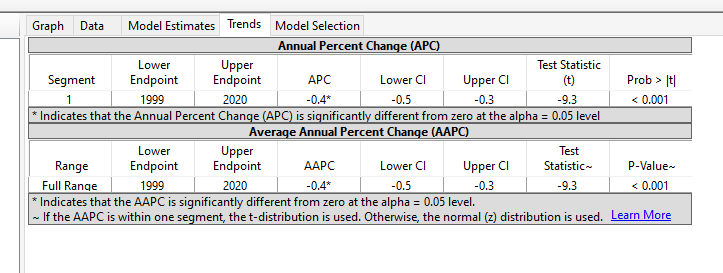


**Supplementary Table 2b.** Temporal trends of bladder cancer mortality by gender.


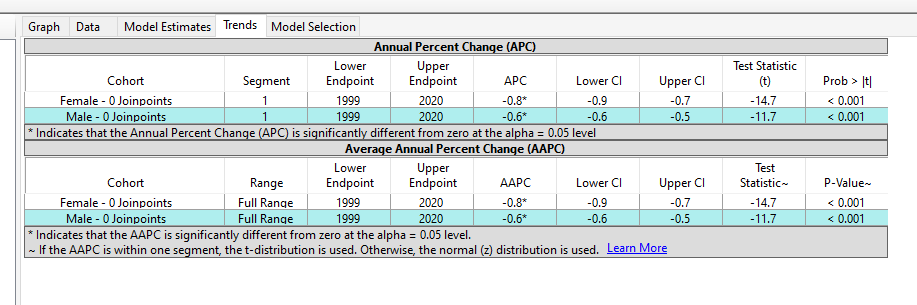


**Supplementary Table 2c.** Temporal trends of bladder cancer mortality by race.


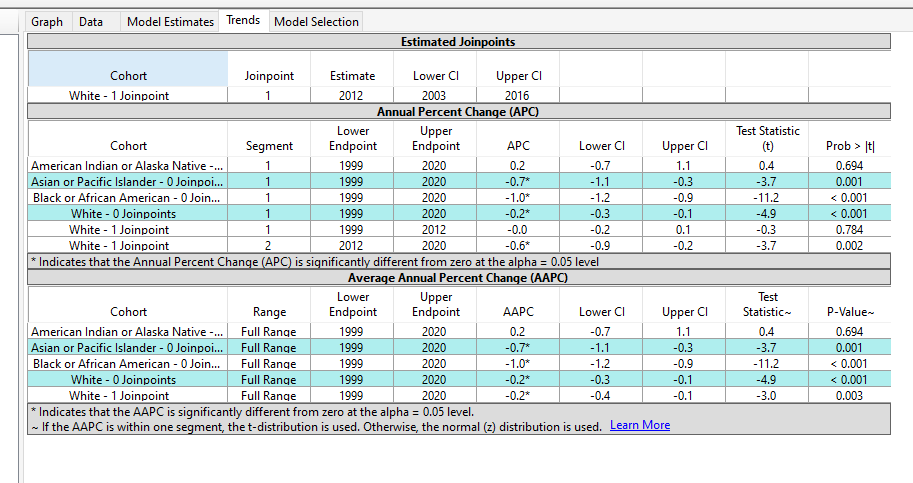


**Supplementary Table 2d.** Temporal trends of bladder cancer mortality in metropolitan areas.


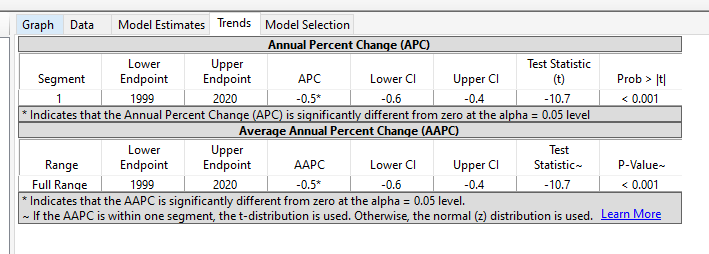


**Supplementary Table 2e.** Temporal trends of bladder cancer mortality in non-metropolitan areas.


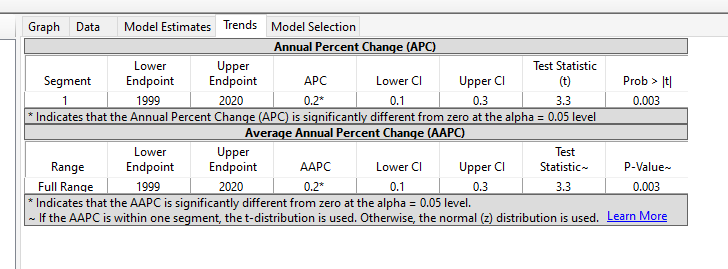


**Supplementary Table 3a.** Temporal trend of kidney cancer mortality in the US.


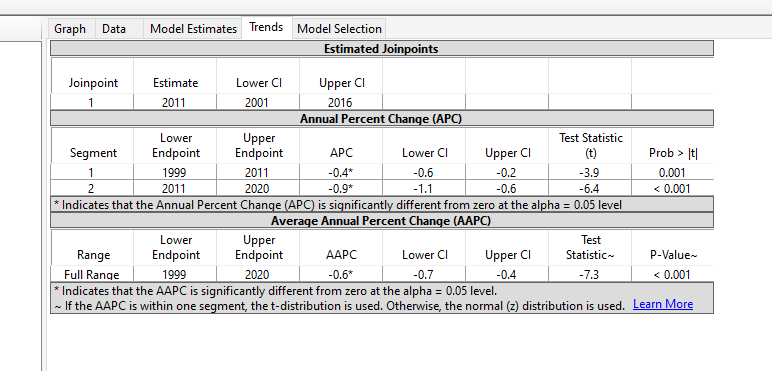


**Supplementary Table 3b.** Temporal trend of kidney cancer mortality by gender.


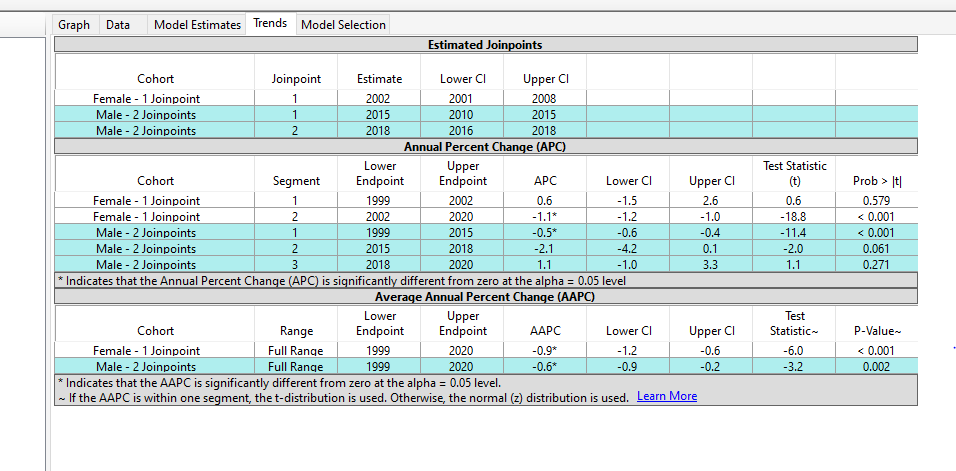


**Supplementary Table 3c.** Temporal mortality trend of kidney cancer mortality by race.


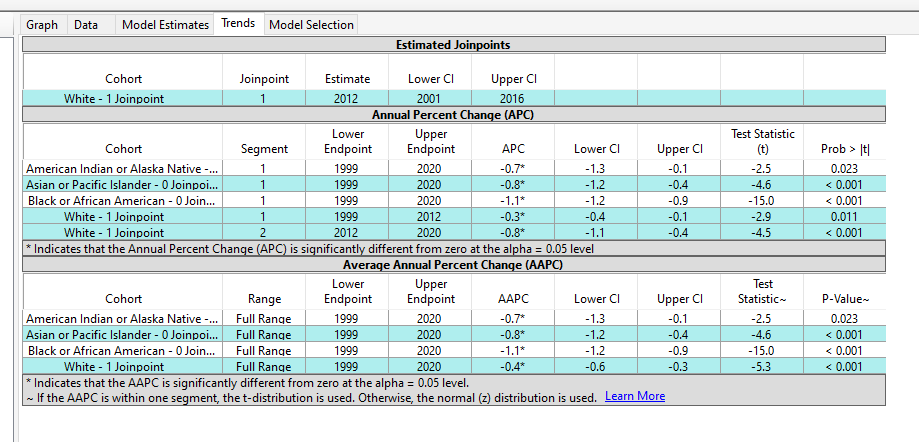


**Supplementary Table 3d.** Temporal trend of kidney cancer mortality in metropolitan areas.
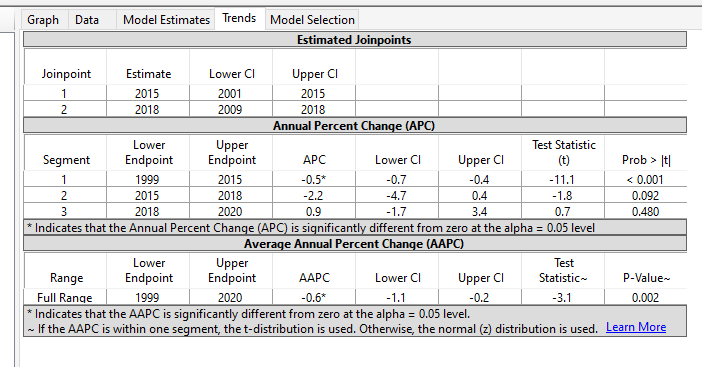


**Supplementary Table 3e.** Temporal trend of kidney cancer mortality in non-metropolitan areas.


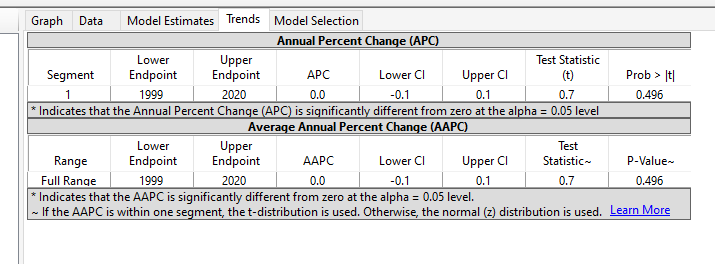


**Supplementary Table 4a.** Temporal trend of overall prostate cancer-related mortality in the US.


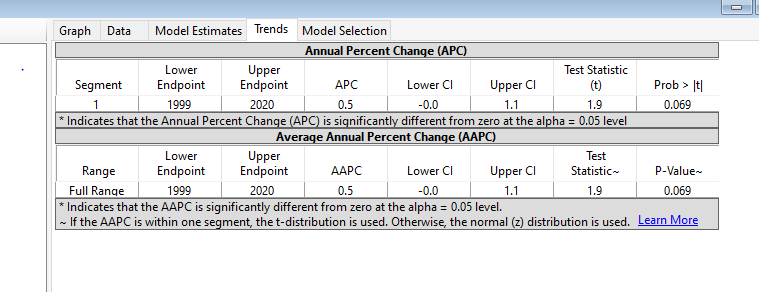


**Supplementary Table 4b.** Temporal trends of testicular cancer mortality by race.


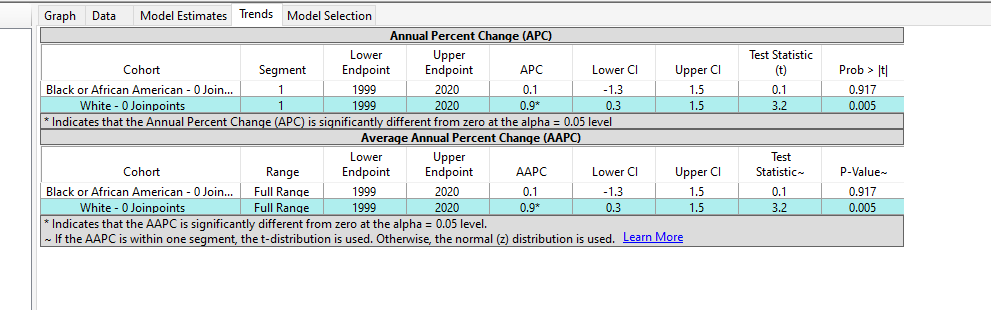


**Supplementary Table 4c.** Temporal trends of testicular cancer mortality in metropolitan areas.


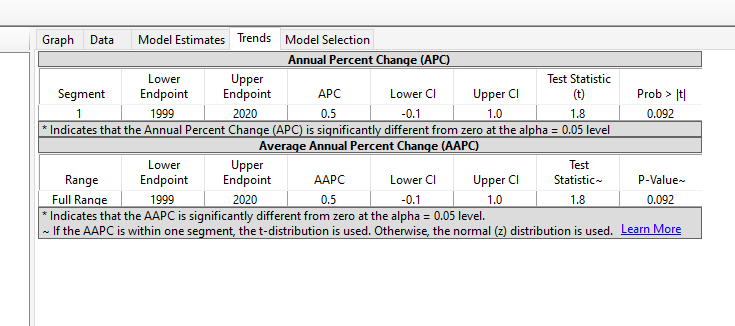


**Supplementary Table 4d.** Temporal trends of testicular cancer mortality in non-metropolitan areas.


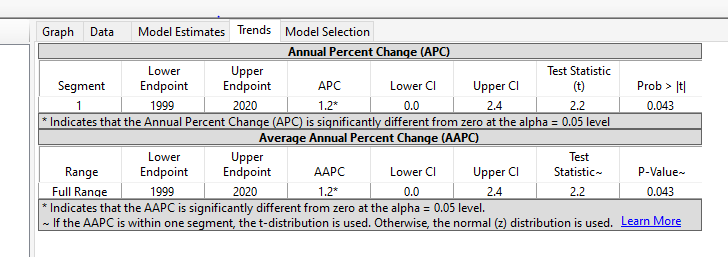


**Supplementary Figure 1. Geographic disparities in prostate cancer mortality in the US.**


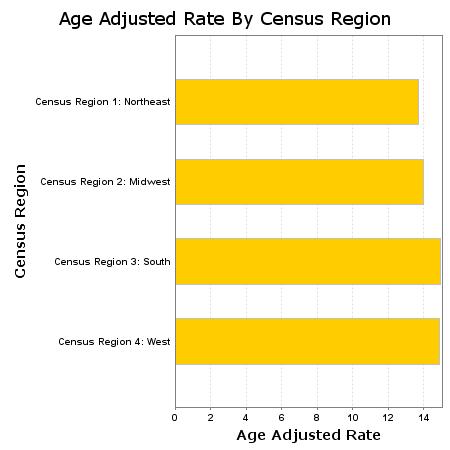


**Supplementary Figure 2. Age adjusted mortality rate by state for prostate cancer in the US.**


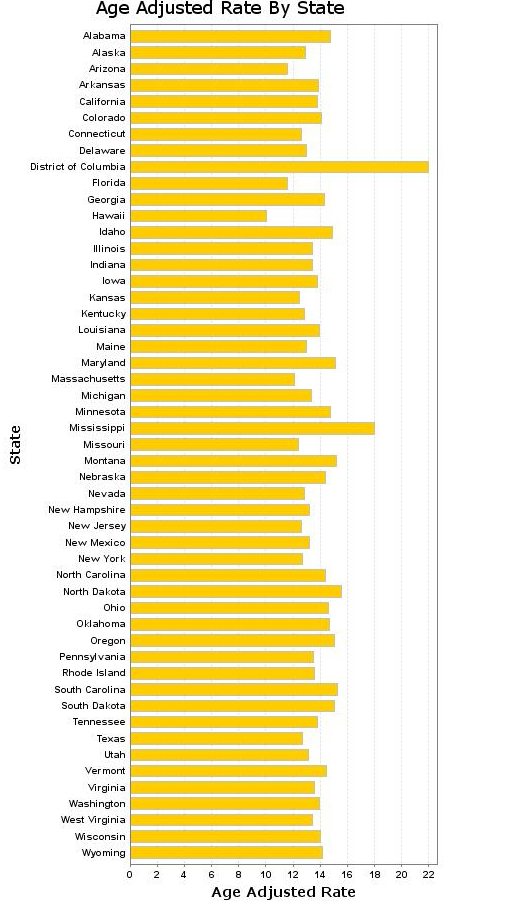


**Supplementary Figure 3. Geographic disparities in bladder cancer mortality in the US.**


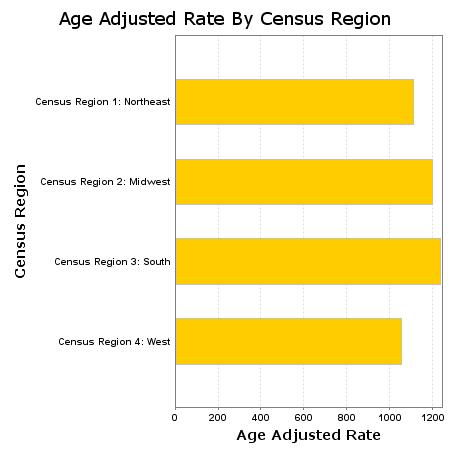


**Supplementary Figure 4. Age adjusted mortality rate by state for bladder cancer in the US.**


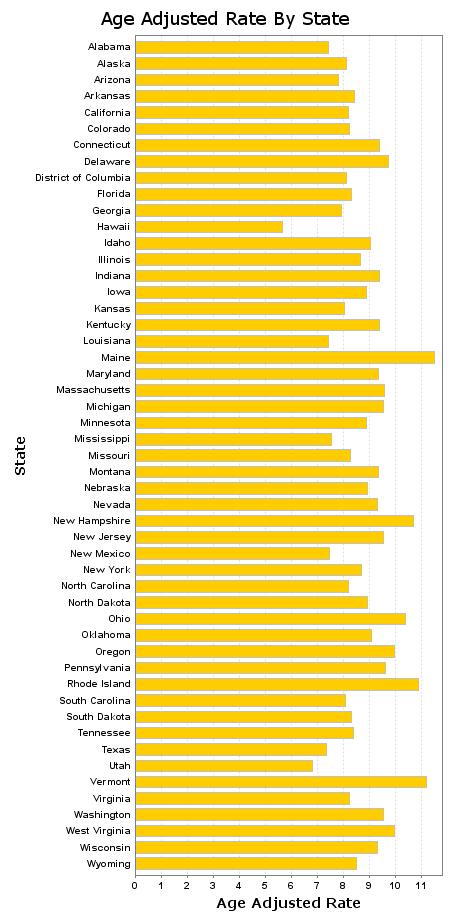


**Supplementary Figure 5. Geographic disparities of kidney cancer mortality in the US.**


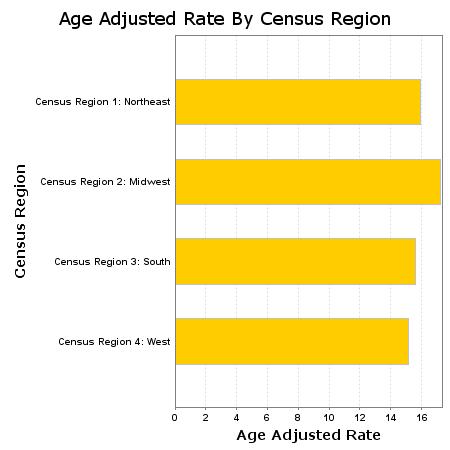


**Supplementary Figure 6. Age adjusted mortality rate by state for kidney cancer in the US.**


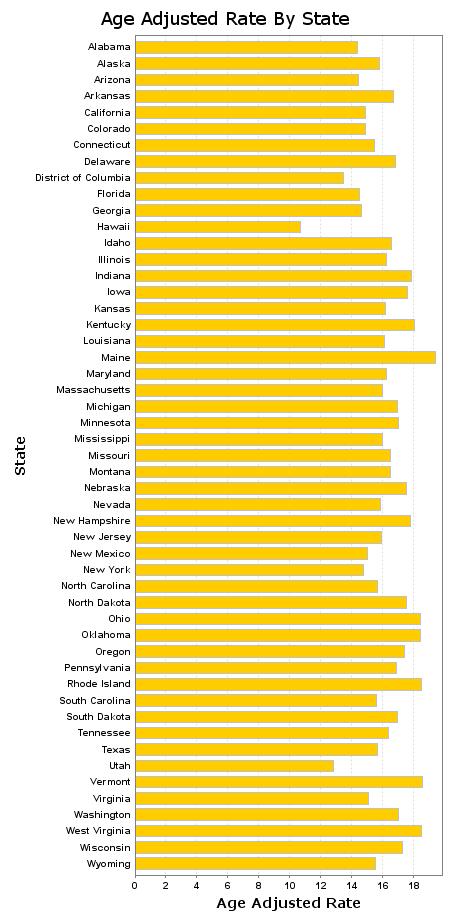


**Supplementary Figure 7. Geographic disparities of testicular cancer-related mortality in the US.**


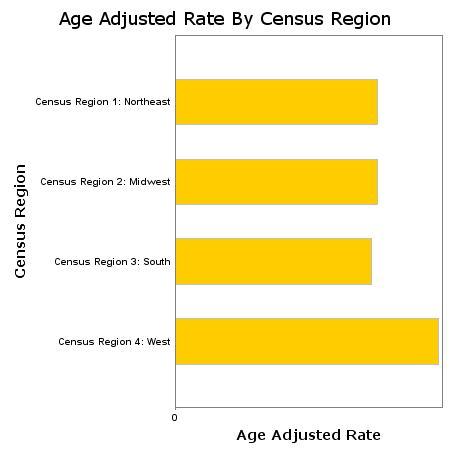


**Supplementary Figure 8. Age adjusted mortality rate by state for testicular cancer in the US.**


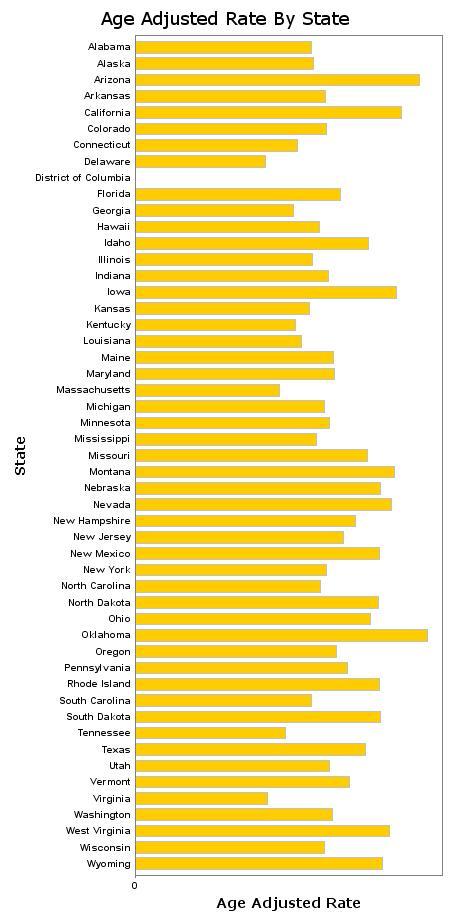

Supplement: Supplementary file 1 [file Data_Sheet_1.docx]
